# Sleeping with the enemy: Maintaining ASF-free farms in affected areas

**DOI:** 10.3389/fvets.2022.935350

**Published:** 2022-09-13

**Authors:** Huybert Groenendaal, Solenne Costard, Francisco J. Zagmutt, Andres M. Perez

**Affiliations:** ^1^EpiX Analytics, Fort Collins, CO, United States; ^2^Center for Animal Health and Food Safety, College of Veterinary Medicine, University of Minnesota, St. Paul, MN, United States

**Keywords:** African Swine Fever, epidemiology, control, biosecurity, risk analysis

## Abstract

African Swine Fever (ASF) continues to spread worldwide, with very limited eradication success in countries where the disease affects domestic pig populations. Various biosecurity tools exist to reduce the on-farm risk incursion of ASF and other diseases. However, their focus on overall biosecurity scores and benchmarking results in recommendations that are not always cost-effective. We propose to apply a risk analysis approach that actively involves farmers and farmworkers in identifying their weakest links in biosecurity and corresponding mitigation efforts. Furthermore, the approach's focus on describing and understanding pathways of introduction and/or spread specific to individual farms creates buy-in from producers for investing in biosecurity measures and improving compliance.

## Introduction

African Swine Fever (ASF) is a disease of domestic and wild swine, caused by the ASF virus (ASFv), which results in far-reaching losses for affected countries and regions. Since 2007, ASFv has widely spread from Sub-Saharan Africa through extensive areas of Russia, Europe, and Asia (1), and was recently introduced into the Americas (2), resulting in an unprecedented spread of pandemic proportions (3).

Many reasons jeopardize ASF control, most notably long ASFv survival times in multiple substrates, circulation of ASFv in wild boar, and absence of commercial vaccines (4). Due to those challenges, since the recent expansion of the disease in 2007, no country has been able to eradicate ASF when their domestic swine population was affected (1). Acknowledging that control and eradication will be onerous and time-consuming for many countries, the challenge of the swine industry in affected areas is to remain free in an ASFv-infected environment, and be able to produce and commercialize pigs, while the epidemic is being controlled. This situation is not new as, for example, ASF was endemic in Spain between 1960 and 1995, and yet during that extended period the country's industry was able to expand (5). More recently infected countries, such as Russia, which has been ASF-infected for almost 15 years now, have also developed policy intended to mitigate risk and sustain business continuity in an endemic setting.

When ASF elimination is not a realistic objective in the short or medium-term, it is important to direct resources and promote public private partnerships to manage disease risk in a way to mitigate disease impact compatible with production, while sustaining and protecting free units (e.g., companies, farms, barns).

Prerequisite for protecting ASF-free units in an affected area is the design and implementation of high biosecurity standards. Biosecurity has been a critical component of ASF risk management efforts worldwide, such as compartmentalization of individual farms in South Africa (6), the Secure Pork Supply Plan for continue of business in the US (7), as well as the recently proposed Partitioning approach (8). Noteworthy, however, given the multiple routes through which the ASFv may enter free barns, farms, or systems, arguably, the strength of any biosecurity plan and implementation would be as strong as its weakest link(s). That is, even though biosecurity may be sufficiently high for most potential pathways of entry, the overall biosecurity of the farm is still low if a single critical pathway is insufficient to mitigate the risk for disease introduction, consequently impairing the effectiveness of producers' overall biosecurity investment.

Risk analysis is an approach to estimate the probability of occurrence and impact of unwanted outcomes (the risks) and manage those risks. Risk analysis includes four main components, namely, hazard identification, risk assessment, risk management, and risk communication. Among those four components, risk assessment focuses on the systematic, transparent, and repeatable evaluation of risks and corresponding mitigation, whereas risk communication is a multidimensional, iterative process that ensures that relevant stakeholders are involved in the other three components. Here, we propose to follow risk analysis principles to undertake self-assessments of biosecurity measures, identify weakest links, and prioritize the implementation of biosecurity measures. The approach can be implemented to prevent the introduction of disease into a free farm or a group of farms within a compartment (external biosecurity), or into free rooms or barns post-ASF introduction onto a farm (internal biosecurity) as part of a Partitioning approach (8).

We first review examples of existing resources to evaluate biosecurity in swine farms in relation to ASF, and then discuss their strengths and pitfalls, and advocate for more actively involving farmers and help creating a biosecurity mindset. A risk analysis approach complements existing biosecurity tools, by describing pathways of ASF introduction to identify and prioritize farm biosecurity measures. Finally, we discuss how this approach is expected to help with prioritizing farm-specific biosecurity investments, more consistency between mitigation measures, and increased adoption and compliance. The perspective presented here will contribute to enhance industry and public authority preparedness with the goal of mitigating the impact of ASF in infected areas.

## Existing “ready to use” resources to evaluate farm biosecurity against ASF

Multiple biosecurity guidelines and assessment tools have been developed by government agencies, academia, and industry. Although quite comprehensive, those guidelines are fairly generic in terms of the scope as defined by the diseases targeted (the “what”), the justification for the recommended practices (the “why”), and the best practices for actual implementation (the “how”). As a result, the tools typically produce scores that serve as a proxy measure of “situational awareness,” but lack detailed guidance as to where to start, what to prioritize under constrained resources (financial, labor, or other), or how to be consistent in the implementation of multiple alternative measures to enhance biosecurity. Most typically, assessment tools aim to provide a “score” indicating how well a given farm is doing with regards to biosecurity practices in comparison to other farms within a company or region. They can be used either for self-assessment, such as the SPS checklists (7), Biocheck.UGent™ (9), BioPorc-RD (10), or as part of an audit like in the 1000-point biosecurity assessment of the PIC BioShield program (11). Existing tools also typically rely on reported practices rather than observations. A detailed overview of available biosecurity tools is available elsewhere (12). Briefly, biosecurity assessment tools broadly fall into two categories:

1. Biosecurity scoring tools derived from expert's epidemiological knowledge of the disease(s) of interest, as well as expert opinion. This approach is typically used to evaluate biosecurity related to foreign animal diseases (FADs). Although such tools may be based on risk assessment (13, 14), they are not data-driven (relevant data are typically unavailable, especially for FADs). Rather, the pathways of entry into and/or spread within a farm, set of criteria and weights applied to obtain the overall biosecurity estimate or score are based on expert(s) perception of their relative importance, and of the efficiency of biosecurity measures. Because they are “theoretical” in nature, they tend to include extensive criteria and recommendations, covering all potentially relevant aspects of biosecurity.
2. Data-driven tools. In this group of tools, outbreak data for endemic diseases are analyzed to quantify risky or protective farm management practices, which are then used to inform the biosecurity recommendations. Although developed based on data from endemic diseases, the resulting biosecurity criteria may be applicable to epidemiologically similar FADs.

Most biosecurity tools from both categories provide an overall score accompanied with some visualizations (e.g., spider charts) that highlight differences in scores between areas of farm management, within a farm or larger organization, or comparing biosecurity to other farms (i.e., benchmarking). Although useful in providing a “picture” (or a one-time, cross-sectional assessment) of the likelihood of farm or system for becoming infected by a disease or group of diseases (as approximated by the level or degree of biosecurity protocols in place), such approaches have the following shortcomings:

1. First, a biosecurity system is only as strong as its weakest link (15, 16). This key notion is often lost when providing an overall score. For example, let us imagine a biosecurity assessment that considers 5 management areas, scored on a scale of 0–10. A farm that consistently performs 8/10 across 5 management areas gets the same overall score as a farm that scores 9/10 across 4 management areas and 4/10 for the remaining area. Yet, while the former farm is consistent in their level of biosecurity the latter farm jeopardizes the work that it does in several areas by very poor biosecurity practices in one area. Thus, existing tools focusing on overall scores, or benchmarking, fall short of helping producers identify their weakest links and prioritize their improvement efforts. Moreover, both because of the criteria considered and because scoring is based on self-reported practices, biosecurity tools typically do not consider how well biosecurity practices are implemented. Recently, data-driven tools have improved on some of the above limitations, as they provide tailored recommendations to individual farms, helping them understand their strengths and weaknesses and prioritize areas of improvement. However, such tools have typically been developed, at least initially, for endemic diseases (e.g., Rabapp for PPRSv), and their applicability to FADs assumes equivalent disease dynamics and/or requires some sort of adaptation for FADs (17).
2. Second, the biosecurity scores are not necessarily explicitly tied to the mechanisms of potential entry of FADs and specific practices that need improvement, and thus can fail to provide a rationale or “buy-in” to the producer and farm staff for implementing recommended practices, especially if costly or time-consuming. The success of any biosecurity effort depends on high compliance in the implementation of the measures. Creating a clear link between mitigation measures and their intended risk reducing purpose is key to create a “biosecurity mindset.”
3. Finally, in most biosecurity tools reviewed, the emphasis is on external biosecurity, while only a few (e.g., Biocheck.UGent) differentiate external vs. internal biosecurity scores. Addressing “internal” biosecurity helps prevent the spread of the disease on the farm and facilitate control to ultimately mitigate disease impact, which can be critical for continuity of business of multi-site swine premises in the face of a FAD epidemic. This is particularly important for ASF given the difficulty in eradicating it from affected regions.

## Using risk analysis for identifying mitigation measures, and developing and improving a biosecurity plan

The World Organization for Animal Health (WOAH, formerly OIE), defines the risk assessment component of risk analysis as the “evaluation of the likelihood and the biological and economic consequences of entry, establishment and spread of a hazard” (18), while risk communication is the “interactive transmission and exchange of information and opinions throughout the risk analysis process.” And Grabill and Simmons (19) argue that an effective risk analysis approach necessitates that “researchers work with audiences in the construction of knowledge (e.g., risk)” to create awareness and identify workable remedial actions.

**Figure 1:**
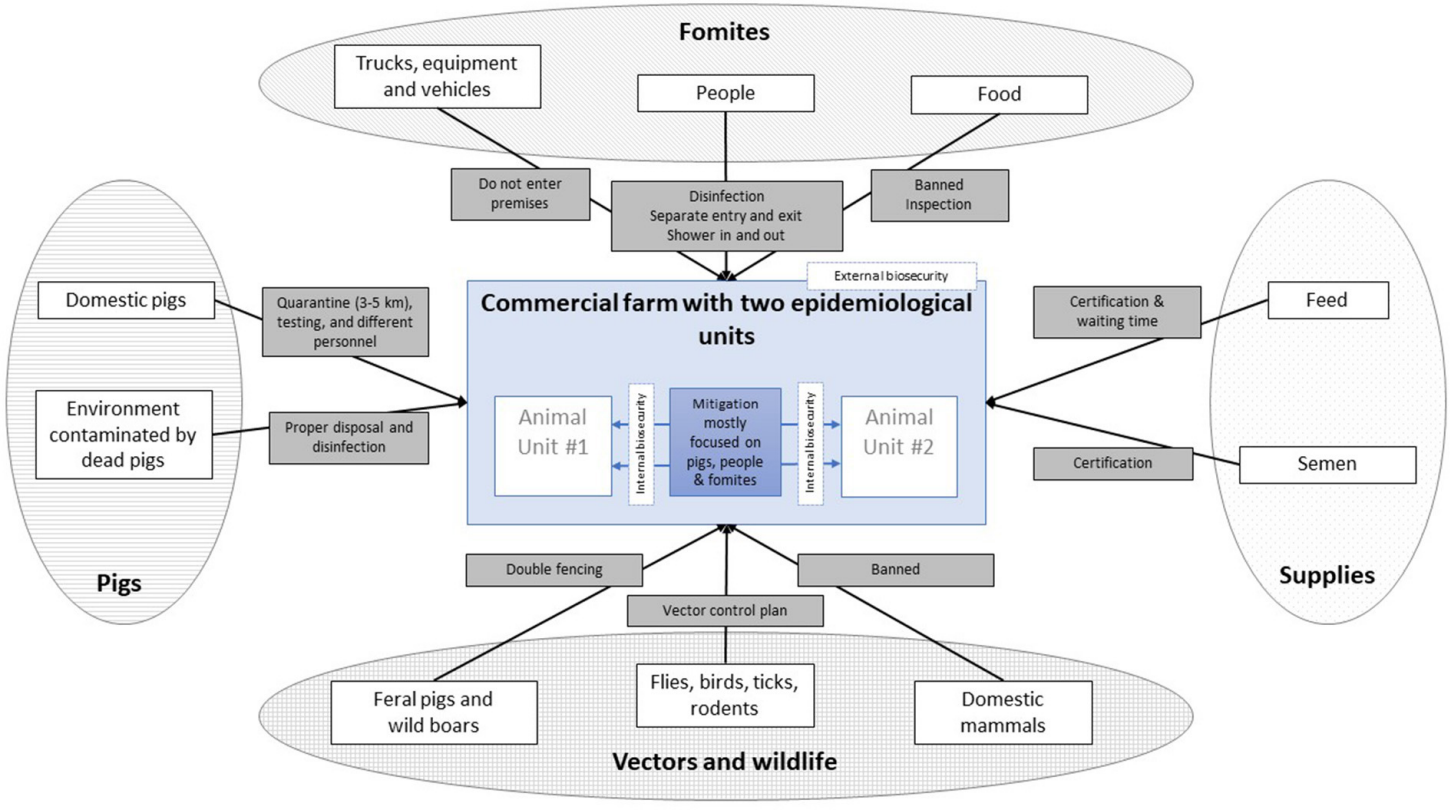
Possible pathways (white boxes, *n* = 10) of introduction of African Swine Fever (ASF) into commercial pig farms, grouped by the primary source of infection (ovals, *n* = 4) and indicating proposed mitigation measures for each of them (gray boxes, *n* = 10).

Here, the audience would be the farmers, ASFv would be the hazard of interest, and the risk, i.e., its entry into farms (or spread within a farm), may take place through a variety of pathways. Figure 1 depicts a simplified diagram of such potential pathways, grouped by the primary source of infection. A pathway represents the “steps” (or events) needed for the virus to be introduced onto a farm. Preventing one, or multiple, of such steps from occurring will result in a mitigation of the risk through that pathway. The likelihood associated with each pathway may be evaluated by reviewing the chain of events that are required for ASF to spread. For example, for ASF entering a farm through food for human consumption, the food will need to be (1) infected with ASF, (2) taken into the farm, and (3) fed to the pigs intentionally or unintentionally. One or multiple mitigation options could then be conceived for each of such pathway events (see gray boxes on Figure 1 - e.g., workers are not allowed to bring their own food, and all food from the cafeteria is incinerated), and their effect estimated while considering compliance.

The aim here is to use pathways to evaluate the relative risk reduction from implementing alternative enhanced biosecurity measures. Indeed, the numeric value of the current risk is not as useful from a management perspective as is the opportunity to identify biosecurity gaps that may be effectively addressed. Consequently, the focus should be on the identification all possible pathways and the identification of nodes, or steps, that could be targeted to further mitigate their respective risk. Thus, the focus is on involving farmers in identifying and describing pathways and determining corrective farm-specific interventions. Increasing producers' understanding of risk pathways and events, having them “think as a virus” (6) by identifying the different steps it would need to get introduced to the farm, and critically thinking through potential risk mitigation efforts will help uncover the “weakest” links (i.e., the greatest risk event within each risk pathway) of the “weakest chains” (i.e., pathway(s) that poses the greatest risk), and help prioritize potential interventions in terms of feasibility, affordability and perceived impact on risk, for a given (typically constraint) producer's budget.

Consequently, the key features of the proposed biosecurity approach, compared to existing tools, include:

- A risk analysis approach focusing on participation of producers and critical thinking through the various disease introduction pathways and farm specific circumstances, rather than on a more formulaic scoring tool and/or comparison with other producers,
- Using 'relative risk scores' to compare disease pathways, and nodes within pathways, so that the overall weakest links can be identified on individual farms, with the ultimate objective of prioritizing correcting measures.

The use of graphical displays for risk communication (e.g., Figure 1), as well as the frequent and continuous reviewing and reporting of risk threats, both part of the proposed approach, have been suggested to increase biosecurity compliance in livestock facilities (20). In addition, “recognizing on-farm biosecurity as practices of bio-secure farming care offers a new way of engaging, motivating and encouraging producers to manage and contain diseases on farm” (21). This is critical when governments increasingly devolve biosecurity governance such as ASF control options, to the farming industry, such as such as compartmentalization efforts in South Africa (22) and Canada (23), and the Secure Pork Supply initiative in the US (7).

## Using the proposed approach to foster a culture of biosecurity

As emphasized by Kotze (6), to successfully manage ASF in an affected area, it is important to “think like a virus” and understand how it could penetrate a farm. A key benefit of using a risk analysis approach is that it works with producers in critically thinking about their specific situation and identifying practical solutions for mitigating the “weakest links.” This contrasts with “black box” approaches that focus on producing biosecurity scores against benchmarks. Guiding questions may be formulated to producers so they can reflect on the relative level of biosecurity achieved (and, potentially, the remaining risk) for each of these pathways. To illustrate this application, an evaluation of a hypothetical farm is shown in Table 1. Two specific risk pathways (an external and an internal pathway) were formulated, based on the authors' observations in multiple ASF-infected countries. For both, three risk events were identified, each of which needs to occur for ASF to spread through the corresponding pathway. The results of the evaluation of alternative mitigations would depend on the farm-specific situation. A full cost-effectiveness analysis would be difficult to complete (24), and instead, factors affecting feasibility, total expenses, affordability, farm layout and workforce, and relative effectiveness should be considered (25). Examples of such factors are also listed in Table 1.

**Table 1:** Evaluation of various fomite-related pathways for introduction of African Swine Fever (ASF) into a hypothetical multi-site commercial sow farm in an endemic environment, indicating, for each of them, areas in which mitigation measures may be implemented.

**Risk events/steps**	**Possible mitigation to address “risk event”**	**Considerations for cost and effectiveness evaluation**
**Example pathway #1: ASF entering premises due to external truck (“external biosecurity”)**		
1. Trucks containing infected materials before entering the farm	Requiring disinfecting of trucks before entering farm	Effectiveness depends on how well trucks are disinfected. Feasibility likely depends on costs and willingness of truck owners/suppliers
2. Truck entering premises	No external trucks entering the premises	Possibly very effective, but may require substantial investment in equipment and infrastructure
3. Infected materials getting exposure to pigs	Cleaning the drop-off or loading area after truck enters premises	Operational costs relatively now. However, given the long-survival time of ASF, effectiveness of this mitigation is likely not high
**Example pathway #2: ASF spreading between units through animal movements (“internal biosecurity”)**		
1. Animals in sourcing units are infected	Increase the external biosecurity to reduce the probability of animals getting infected in the first place	By increasing external biosecurity, the farm will also reduce the probability of spread between units within the farm
2. Infected animals to be moved to another unit	Testing animals before movement between units, and/or quarantine of animals before movement between units	Either of the two risk mitigation options would reduce the risk of spread between units. Modeling could help determine which option(s) is/are most cost-effective
3. Infected animals need to be exposed to animals in second (ASF-free) unit	All-in-all-out system	By moving a group of animals to an empty unit, the producer could avoid the potential spread of ASF between units. Feasibility depends on farm situation and may require capital investments.

There are multiple ways in which this approach can complement existing tools to assist the objective of sustaining ASF-free farms and units in an affected area. First, if applied to external biosecurity, the approach may be part of the efforts to create an ASF free compartment to support trade (6), or to support continuity of business (7). Alternatively, if applied to both external and internal biosecurity (i.e., to mitigate the risk for ASFv spread within a company or within units in a multi-site premises) the approach can be part of a Partitioning approach intended to protect free epidemiological units within an affected system (8). Then, a risk analysis approach promoting critical thinking through disease introduction pathways, and focusing on “weakest links”, is especially useful as an underpinning of creating a biosecurity culture (6), and supporting continuous biosecurity improvements. For example, farms could undergo yearly third-party biosecurity audits, as is currently done for commercial pork producers in South Africa (6) or poultry producers in the US (26). This repeated, iterative approach would be especially useful in the long-term, as it would allow focusing on changes between audits, and can be more efficiently updated regularly compared to existing tools – while accounting for the wide range of producers' specific circumstances, including the specific farm context with regards to (local) ASF infections. Given that the approach relies on guided self-assessment and requires critical thinking through potential risk pathways and possible mitigation strategies, it also fosters buy-in from farm workers and creates a culture of biosecurity.

Finally, the approach can also help producers to check for consistency (and possible gaps) in their mitigation measures and thus prioritize additional biosecurity improvements. Specifically, the approach here can be particularly helpful if producers have some flexibility and discretion about which exact biosecurity measures to take because, for example (1) biosecurity measures are unregulated in the country, (2) the producers aim to exceed the minimum level of biosecurity required by regulations, or (3) when a certification program requires improvement of biosecurity over time, and budget constraints require a tiered implementation.

We highlight that our approach can leverage or complement existing biosecurity tools. For example, tools such as BioCheck (9) can provide an overview of potential risk pathways, while the current approach can focus on identifying weakest links and pragmatic mitigation options.

## Conclusion

The perspective presented here is complementary to existing biosecurity tools for the critical evaluation of biosecurity on pig farms, using a risk analysis approach with the objective of actively involving producers in identifying and prioritizing the implementation of disease mitigation measures. The approach fosters an awareness of risk pathways and creates a culture of biosecurity (6). Acknowledging that eradication of ASFv is not a realistic option in the foreseeable future for many affected countries, the proposed approach can help the design and sustainable implementation (i.e., cost-effective, progressive and with high compliance) of mitigation strategies with the goal of reducing the impact of disease on free farms and epidemiological units that operate in ASF affected areas. This approach will require more validation and field testing to be broadly implementable in the field. Certain recent examples, such as initiatives implemented in South Africa, suggest the approach can become part of the biosecurity toolbox for enhancing preparedness to mitigate the impact of ASF incursions in free or infected regions.

## Data availability statement

The original contributions presented in the study are included in the article/supplementary material, further inquiries can be directed to the corresponding author/s.

## Author contributions

HG, SC, and AP designed the project and conceived the idea and wrote the first version of the manuscript. All authors provided critical feedback and helped shape the research and manuscript. All authors contributed to the article and approved the submitted version.

## Funding

This work was supported by Section 108 Foreign currency program [Federal Award Identification number 108-2019-05] from the USDA FAS.

## Conflict of interest

SC, FZ, and HG are employed by EpiX Analytics LLC. All authors declare that the research was conducted in the absence of any commercial or financial relationships that could be construed as a potential conflict of interest.

## Publisher's note

All claims expressed in this article are solely those of the authors and do not necessarily represent those of their affiliated organizations, or those of the publisher, the editors and the reviewers. Any product that may be evaluated in this article, or claim that may be made by its manufacturer, is not guaranteed or endorsed by the publisher.

## References

[1] OIE. Global situation of African Swine Fever. (2020). Available online at: https://www.oie.int/app/uploads/2021/03/report-47-global-situation-asf.pdf (accessed January 20, 2022).

[2] Gonzales W Moreno C Duran U Henao N Bencosme M Lora P. African swine fever in the Dominican Republic. Transbound Emerg Dis. (2021) 68:3018–9. 10.1111/tbed.14341 34609795

[3] Ward MP Tian K Nowotny N. African swine fever, the forgotten pandemic. Transbound Emerg Dis. (2021) 68:2637–9. 10.1111/tbed.14245 34499823

[4] Dixon LK Stahl K Jori F Vial L Pfeiffer DU. African swine fever epidemiology and control. Ann Rev Animal Biosci. (2020) 8:221–46. 10.1146/annurev-animal-021419-083741 31743062

[5] Arias M Sánchez-Vizcaíno J Morilla A Yoon KJ Zimmerman J. African swine fever eradication: The Spanish model. African Swine Fever Eradication. (2002) 2002:133–9. 10.1002/9780470376812.ch4c32469923

[6] Kotze J,. Living with ASF in South Africa. (2022). Available online at: https://www.pigprogress.net/health-nutrition/podcast-living-with-asf-in-south-africa/ (accessed January 19, 2022).

[7] Secure Pork Supply Plan,. Self-Assessment Checklist for Enhanced Pork Production Biosecurity: Animals Raised Indoors. (2017). Available online at: https://www.securepork.org/Resources/SPS_Biosecurity_Self-Assessment_Checklist-_-IndoorProduction.pdf (accessed January 20, 2022).

[8] Costard S Perez AM Zagmutt FJ Pouzou JG Groenendaal H. Partitioning, a novel approach to mitigate the risk and impact of African Swine Fever (ASF) in endemic settings. Front Vet Sci. (2022) 8:812876. 10.3389/fvets.2021.812876 35274016 PMC8902292

[9] Gelaude P Schlepers M Verlinden M Laanen M Dewulf J. Biocheck.UGent: a quantitative tool to measure biosecurity at broiler farms and the relationship with technical performances and antimicrobial use. Poultry Sci. (2014) 93:2740–51. 10.3382/ps.2014-04002 25193257

[10] African swine fever risk tool aids Dominican Republic pork producers [Internet]. National Hog Farmer. (2022). Available online at: https://www.nationalhogfarmer.com/news/african-swine-fever-risk-tool-aids-dominican-republic-pork-producers (accessed March 29, 2022).

[11] PIC. Biosecurity Standards for PIC Multiplication Units and Gene Transfer Centers. (2019). Available online at: https://www.pic.com/wp-content/uploads/sites/3/2019/09/PIC-Biosecurity-Standards.pdf (accessed January 20, 2022).

[12] Alarcón LV Allepuz A Mateu E. Biosecurity in pig farms: a review. Porcine Health Manage. (2021) 7:5. 10.1186/s40813-020-00181-z 33397483 PMC7780598

[13] Lewerin SS Österberg J Alenius S Elvander M Fellström C Tråvén M. Risk assessment as a tool for improving external biosecurity at farm level. BMC Vet Res. (2015) 11:171. 10.1186/s12917-015-0477-7 26215281 PMC4515931

[14] Allepuz A Martín-Valls GE Casal J Mateu E. Development of a risk assessment tool for improving biosecurity on pig farms. Prev Vet Med. (2018) 153:56–63. 10.1016/j.prevetmed.2018.02.014 29653735

[15] Johnson C. Pig Health Today: Johnson: Make Your Farm a Biosecurity Fortress on Apple Podcasts. (2022). Available online at: https://podcasts.apple.com/ee/podcast/johnson-make-your-farm-a-biosecurity-fortress/id1272328272?i=1000545679720 (accessed January 19, 2022).

[16] Levis DG Baker RB. Biosecurity of Pigs Farm Security [Internet]. Pork Information Gateway. (2011). Available online at: https://porkgateway.org/resource/biosecurity-of-pigs-and-farm-security/

[17] Machado G. The Rapid Access Biosecurity (RAB) appTM [Internet]. Machado lab. (2022). Available online at: https://machado-lab.github.io/rabapp/

[18] OIE. Glossary. (2021). Available online at: https://www.oie.int/fileadmin/Home/eng/Health_standards/tahc/current/glossaire.pdf

[19] Grabill JT Simmons WM. Toward a critical rhetoric of risk communication: Producing citizens and the role of technical communicators. Tech Commun Q. (1998) 7:415–41. 10.1080/10572259809364640

[20] Merrill SC Moegenburg S Koliba CJ Zia A Trinity L Clark E. Willingness to comply with biosecurity in livestock facilities: evidence from experimental simulations. Front Vet Sci. (2019) 6:e00156. 10.3389/fvets.2019.00156 31214603 PMC6558082

[21] Maye D Chan KWR. On-farm biosecurity in livestock production: farmer behaviour, cultural identities and practices of care. Emerg Top Life Sci. (2020) 4:521–30. 10.1042/ETLS20200063 32909609

[22] Pfeiffer DU Ho HPJ Bremang A Kim Y OIE team. Compartmentalisation Guidelines - African Swine Fever. Paris: World Organisation for Animal Health (OIE) (2021).

[23] Government of Canada CFIA. Canada's proposed National Standards for African Swine Fever Compartments. (2022). Available online at: https://inspection.canada.ca/about-cfia/transparency/consultations-and-engagement/asf-compartments/national-standards/eng/1645140402937/1645140403296

[24] Rojo-Gimeno C Postma M Dewulf J Hogeveen H Lauwers L Wauters E. Farm-economic analysis of reducing antimicrobial use whilst adopting improved management strategies on farrow-to-finish pig farms. Prev Vet Med. (2016) 129:74–87. 10.1016/j.prevetmed.2016.05.001 27317325

[25] Babo Martins S Rushton J. Cost-effectiveness analysis: adding value to assessment of animal health, welfare and production: -EN- -FR- L'analyse coûts-efficacité: une valeur ajoutée apportée à l'évaluation de la santé, du bien-être et de la production des animaux -ES- El análisis de la relación costo-eficacia como valor añadido a las evaluaciones de salud, bienestar y producción animales. Rev Sci Tech OIE. (2014) 33:681–9. 10.20506/rst.33.3.2312 25812198

[26] NPIP. Animal Health. (2022). Available online at: http://www.poultryimprovement.org/default.cfm?CFID=60228&CFTOKEN=4474cb185bbef423-25DC57D8-053A-935D-329F0A87C002EF2D~

